# Thyroid Stimulating but Not Blocking Autoantibodies Are Highly Prevalent in Severe and Active Thyroid-Associated Orbitopathy: A Prospective Study

**DOI:** 10.1155/2015/678194

**Published:** 2015-06-28

**Authors:** E. Kampmann, T. Diana, M. Kanitz, D. Hoppe, G. J. Kahaly

**Affiliations:** ^1^Molecular Thyroid Research Laboratory, Johannes Gutenberg University (JGU) Medical Center, 55131 Mainz, Germany; ^2^Department of Pediatrics, Johannes Gutenberg University (JGU) Medical Center, 55131 Mainz, Germany; ^3^Department of Psychology, Technical University of Darmstadt, 64283 Darmstadt, Germany

## Abstract

The clinical utility of the functional TSH receptor autoantibodies was prospectively evaluated in patients with thyroid-associated orbitopathy (TAO). Ophthalmic, endocrine, and serological investigations were performed in 101 consecutive patients with severe and active TAO. Serum thyroid stimulating (TSAb) and blocking (TBAb) antibody levels were measured with two bioassays using cells that express a chimeric TSH receptor and CRE-dependent luciferase. TSAb results are expressed as percentage of specimen-to-reference ratio (SRR %). Blocking activity is defined as percent inhibition of luciferase expression relative to induction with bovine TSH alone. All 101 consecutively followed-up patients with severe and active TAO were TBAb negative. In contrast, 91 (90%) were TSAb positive of whom 90 had Graves' disease. Serum TSAb levels correlated with the diplopia score (*P* = 0.016), total severity eye score (*P* = 0.009), proptosis (*P* = 0.007), lid aperture (*P* = 0.003), upper lid retraction (*P* = 0.006), keratopathy (*P* = 0.04), and thyroid binding inhibiting immunoglobulins (TBII, *P* < 0.001) and negatively with the duration of TAO (*P* = 0.002). Median serum values of TSAb were SRR% 418 (range 28% to 795%). TSAb, not TBAb, are highly prevalent in severe/active TAO and serum TSAb levels correlate with clinical disease severity.

## 1. Introduction

Thyroid-associated orbitopathy (TAO) is mostly associated with Graves' hyperthyroidism [1]. Typical and frequently observed signs include eyelid retraction and exophthalmos [2]. The activity of TAO is generally determined by the clinical activity score (CAS) [3], consisting of seven different orbital signs and symptoms. For each positive variable one point is scored with a total sum of 7. Werner proposed the NOSPECS score for assessing disease severity, a score that was revised in 1977 [4]. A disease-specific quality of life questionnaire has been developed [5], which has shown that this disease reduces quality of life by negatively affecting perception of appearance and functionality [6]. The therapeutic options for TAO vary and should be determined individually for each patient, preferably in a multidisciplinary thyroid-eye clinic [7]. The pathogenesis of TAO is not yet fully elucidated but is most likely multifactorial involving an underlying series of autoimmune processes [8].

The presence of autoantibodies to the TSH receptor (TSH-R) is a parameter used for diagnosis of Graves' disease (GD). Functional TSH-R autoantibodies can be divided in either blocking (TBAb) [9] or stimulating (TSAb) antibodies. Both TSAb and the thyrotropin binding inhibitory immunoglobulins (TBII) reduce the rate of false anti-TSH-R negative patients with GD [10]. Furthermore, levels of TSAb discriminate between responders and nonresponders during antithyroid drug treatment [11]. The TSH-R is also expressed in the orbit and on the surface of the lacrimal cells [12, 13]. Ethnic background (Caucasians or Asians) may have an effect on the correlation of TSH-R antibodies with TAO [8, 14–17]. Previous studies have shown that TSAb influence the course of TAO [18, 19], but the role of TBAb is still a matter of debate due to scarce data.

Therefore, this prospective study aimed to evaluate the clinical relevance and diagnostic role of the functional (TSAb and TBAb) TSH-R autoantibodies in a large group of consecutive patients with severe and active TAO regularly followed up and treated at an academic tertiary referral multidisciplinary orbital center with a joint thyroid-eye clinic.

## 2. Methods

### 2.1. Subjects

This prospective study was approved by the local Ethical Committee and all patients gave their informed written consent. We followed the tenets of the declaration of Helsinki. Consecutive patients with severe and active TAO aged 23–75 years followed up at our institution between 2009 and 2014 were included. All patients were euthyroid at the time of inclusion in the study, as indicated by serum concentrations of free T4, free T3, and baseline TSH within the normal range. 36 patients were currently on antithyroid drugs, 47 were on levothyroxine, L-T4, either as a monotherapy or in combination with T3 subsequent to thyroid surgery or radioactive iodine treatment, and 17 patients were not receiving any medication. Diagnosis of TAO was based on clinical criteria according to the consensus statement of the European Group on Graves' orbitopathy (EUGOGO). Clinical disease activity and clinical severity were evaluated according to the clinically activity score (CAS) [3] and the modified NOSPECS score [4].

### 2.2. Bioassay for Measurement of Thyroid Stimulating Antibodies (TSAb)

Serum TSAb levels were measured with a novel FDA-cleared cell-based bioassay (Thyretain, Quidel Corp., San Diego, CA, USA) according to the manufacturer's instructions. This assay utilizes Chinese hamster ovary cells (chimeric-CHO-luc) constitutively expressing a chimeric TSH-R and a firefly luciferase gene downstream of a promoter containing cAMP responsive elements as previously described [20]. Briefly, chimeric-CHO-Luc cells were seeded and grown to a confluent cell monolayer in 96-well plates for 15 to 18 hours. Patient serum samples, positive, reference, and normal controls were diluted 1 : 11 in reaction buffer and added to the cell monolayers, and each plate was incubated for three hours at 37°C in 5% CO_2_. Subsequently, the chimeric-CHO-Luc cells were lysed, and the relative light units were quantified in a luminometer (Infinite M200; Tecan GmbH, Crailsheim, Germany). The samples were measured in triplicate and reported as the percentage of specimen-to-reference ratio (SRR%).

### 2.3. Thyroid-Blocking Antibody (TBAb) Bioassay

Serum TBAb levels were measured with a novel cell-based bioassay as previously described [9]. Blocking activity was defined as percentage inhibition of luciferase expression relative to induction with bovine TSH alone.

### 2.4. Thyrotropin-Binding Inhibitory Immunoglobulin (TBII) Assay

Serum TBII levels were measured using commercially available kits according to the manufacturer's instructions (Thermofisher, Brahms Diagnostic, Berlin, Germany).

### 2.5. Statistical Analysis

The statistical analysis was computed using SPSS (SPSS, Version 18, SPSS Inc., Chicago, IL, USA). The descriptive statistic was performed by calculation of the mean, median, standard deviation, and minimum and maximum values. The Mann-Whitney *U* test was performed to detect statistically significant differences of the mean ranks between two groups with respect to an ordinal or interval type variable. If the independent variable included more than two groups, the differences of the mean ranks were calculated by the Kruskal-Wallis test, another nonparametric test. When there were two interval or ratio type variables or the ordinal type variable contained a sufficient number Spearman's rank correlation coefficient was calculated. A statistical significance was assumed when the *P* value was <0.05.

## 3. Results

### 3.1. Demographic Data

Demographic and clinical parameters are shown in Table 1. The female-to-male ratio was 3 : 1. A total of 70 patients had values above the cut-off of 10 mm for palpebral aperture in at least one eye. Also, 63 patients showed proptosis values above the cut-off of 20 mm (for Caucasians) in at least one eye. Asymmetric exophthalmos or proptosis was defined as the difference between the two eyes of at least 3 mm.

### 3.2. Serological and Immunological Data

**Figure 1:**
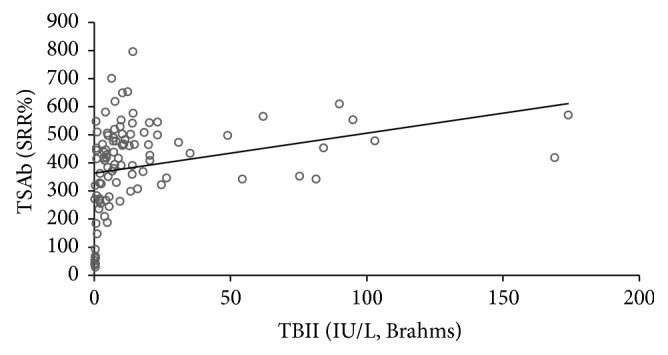
Correlation between serum TSAb and TBII levels. Spearman's rho = 0.538; *P* < 0.001.

**Figure 2:**
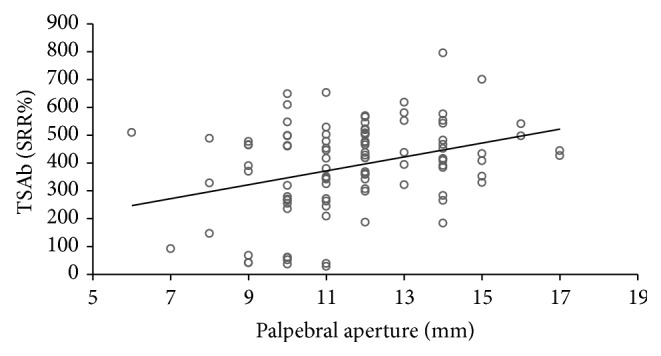
Correlation of serum TSAb levels with palpebral aperture. Spearman's rho = 0.29; *P* = 0.003.

**Figure 3:**
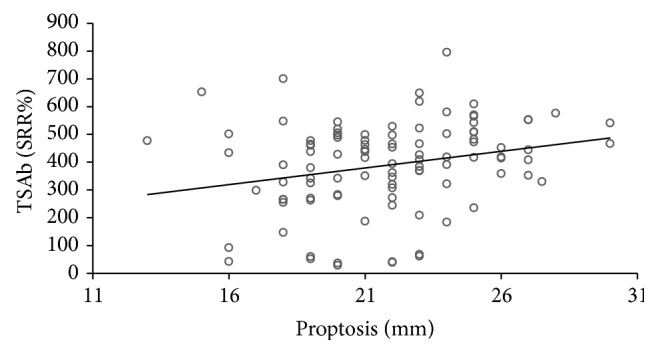
Correlation of serum TSAb levels with proptosis. Spearman's rho = 0.27; *P* = 0.007.

**Figure 4:**
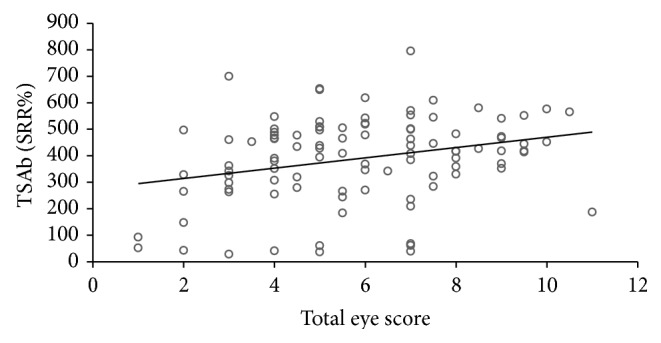
Correlation of serum TSAb levels with the total severity eye score. Spearman's rho = 0.26, *P* = 0.009.

**Figure 5:**
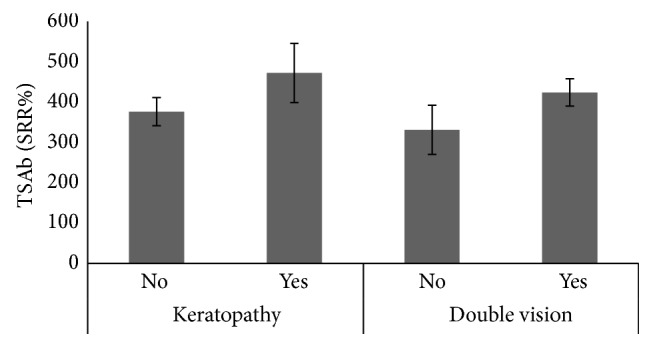
Right panel: mean TSAb levels in patients with or without diplopia (Mann-Whitney *U* test; *P* = 0.023); left panel: mean TSAb levels in patients with or without corneal lesions (keratopathy, Mann-Whitney *U* test; *P* = 0.042).

All 101 consecutive patients with severe and active TAO were TBAb negative. In contrast, 91 (90%) were TSAb positive. All TSAb positive samples were also positive (negative inhibition) in the blocking assay with negative inhibition values > minus 100%. 90 of 101 TSAb positive patients had GD, while only one had Hashimoto's thyroiditis (HT). The other three HT patients were TSAb negative as were four GD patients who had undergone complete thyroidectomy. Additional two patients had been treated with radioactive iodine (seven and ten years prior to presentation) and one patient had taken antithyroid drugs. The duration of TAO negatively correlated with serum TSAb levels (Spearman's rho = −0.303; *P* = 0.002) and TBII (Spearman's rho = −0.296; *P* = 0.003). Serum TSAb levels also strongly correlated with TBII serum levels (Spearman's rho = 0.538; *P* < 0.001; Figure 1) and the maximum palpebral aperture (Spearman's rho = 0.29; *P* = 0.003; Figure 2) and proptosis (Spearman's rho = 0.27; *P* = 0.007; Figure 3). There was a significant difference in the upper lid retraction when the patients were divided into groups according to their respective TSAb value (negative, low, i.e., SRR% 140–279, moderate, i.e., 280–419, and high, i.e., > 419, positive; *P* = 0.006). Subsequent pairwise Mann-Whitney *U* tests showed that patients negative for TSAb scored lower on upper lid retraction. TSAb levels also correlated with a higher clinical severity score (Spearman's rho = 0.260; *P* = 0.009; Figure 4). Furthermore, we observed a higher serum level of TSAb in patients with diplopia (*P* = 0.023; Figure 5, right panel) and corneal damage or keratopathy (*P* = 0.042; Figure 5, left panel).

Although correlations between TSAb and several clinical findings of TAO were found, in this study there was no significant correlation in patients with higher CAS (*P* = 0.797) or with chemosis (*P* = 0.214). In cigarette smokers, the number of pack years showed a negative trend with the CSS (Spearman's rho = −0.252; *P* = 0.05), but there was no significant influence of smoking habits on CAS. Daily cigarette consumption or pack years had no significant impact on levels of TSAb (*P* = 0.686 and *P* = 0.789, resp.) or TBII (*P* = 0.317 and *P* = 0.857, resp.).

## 4. Discussion

This study clearly demonstrates in a large collection of patients with TAO that TSAb are highly prevalent in patients with severe and active orbital disease. This is also the first paper to assess the prevalence of blocking TSH-R autoantibodies in patients with TAO using a newly developed and validated bioassay which is able to measure both blocking and stimulatory activity (i.e., by virtue of observing negative inhibition) [9]. Not a single patient with TAO was positive for TBAb, strongly suggesting that the blocking autoantibodies do not play a major role in the immunopathogenesis of the orbital changes in TAO. However, our results emphasize a putative role of TSAb in the pathophysiology of TAO. Based on these data, as well as those previously published by our group, stimulatory autoantibodies, in strong contrast to TBAb, are valuable and useful biomarkers of TAO. Although causality cannot be proven in this paper, the high correlation of these stimulatory autoantibodies with the presence of severe TAO strongly argues for a functional association.

The correlation curves between TSAb and proptosis and total eye score were weak; nevertheless given that TSAb levels correlate with several signs and symptoms of clinical severity of TAO, it is possible that TSAb may play a role in development of the extrathyroidal manifestations of this complex and systemic disease. It is not unreasonable to speculate that TSAb bind to and stimulate the activity of the TSH-R-expressing target cells in the eye, skin, and bone leading to TAO, thyroid-associated dermopathy, and acropachy. The TSAb effects may be mediated through the TSH-R in orbital tissue and evidence has accumulated that these receptors may be functional. TSAb activation of orbital TSH-R receptors upregulates expression of important proteins in TAO in a similar fashion to that seen by TSH activation [21]. Both supraphysiological doses of TSH and high TSH-R expression on orbital fibroblasts induce adipogenesis and lead to TAO. We thus hypothesize that the production of TSAb is one trigger for the initiation of TAO. Aside from adipogenesis, TSAb may have the potential to upregulate or alter antigens, costimulatory proteins, or other effectors important in TAO. Further support for the role of TSAb in the pathogenesis of TAO comes from animal models showing that a Th2 autoimmune response to the TSH-R may be prerequisite for the development of TAO [22].

In a previous report, serum TSAb levels were significantly higher in patients with TAO and untreated GD compared to those without TAO [17]. In this previous study, logistic regression analysis showed that TSAb levels were independent predictors of TAO. In contrast, no correlation between the binding assay (TBII) and eye disease was found. The prevalence of TAO increased with each incremental quartile of TSAb levels. Furthermore, the odds ratio of TAO was high when TSAb levels were above the median level. In a further study [8], TSAb was the strongest independent predictor of four features of TAO: lid fullness, proptosis, lid retraction, and extraocular myopathy. Also, in a cohort of TAO patients, serum TSAb levels significantly correlated with TAO clinical severity score, but no association was found between TBII levels and TAO scores [23]. With the exception of a trial reporting that both TSAb and TBII levels correlate with CAS [16] these previous studies neither differentiated between activity and severity of TAO, nor examined all the individual symptoms and signs that comprise the CAS.

In our present prospective study, smoking habits did impact neither the clinical phenotype of TAO nor the serum levels of the functional and binding TSH receptor autoantibody levels. This is in contrast to previous reports and to the data published in the consensus statement of the European Group on Graves' orbitopathy [24]. At our institution, we have observed over the last years a significant decrease of the smoking rate in patients with TAO and GD. Furthermore, our data refer to a specialized tertiary referral center with an academic joint thyroid-eye clinic probably differing from the clinical practice. Both the marked reduction of nicotine consumption and the special situation of our specialized center might play a role pertaining to the noted discrepancies. Also and although all patients with TAO had clinically active disease and were widely positive for TSAb with a few exceptions only, there was no positive correlation between serum TSAb/TBII levels and the clinical activity score in the present study. However, this somehow surprising result does not definitely contradict previous reports on the positive association between TSH receptor autoantibody levels and CAS as no patients with inactive TAO were included.

In conclusion, two novel cell-based bioassays for the measurement of functional TSH-R autoantibodies have demonstrated that TSAb, but not TBAb, are widely present in TAO and closely correlate with disease severity.

## Figures and Tables

**Table 1:** Demographic, clinical, and serological data of the 101 consecutively included patients with severe and active TAO.

GD	*N* = 97
HT	*N* = 4
Female/male	*N* = 76/25
Current smokers	*N* = 53
Median age	52 years (range 23–75)
Median duration of TAO	11 months
Median palpebral aperture	12 mm (6–17 mm)
Median proptosis	22 mm (13–30 mm)
Asymmetric proptosis	*N* = 20
Median CAS	4 (3–6)
Median CSS	5.75 (1–11)
Diplopia	*N* = 63 (12 constant)
Upper lid retraction	*N* = 52
Lower lid retraction	*N* = 42
Chemosis	*N* = 27
Corneal lesions	*N* = 14
TSAb	Median SRR% 418 (28–795)
TBII	Median 7.35 IU/l (0.3–174)

GD: Graves' disease.

HT: Hashimoto's thyroiditis.

TAO: thyroid-associated orbitopathy.

CAS: clinical activity score.

CSS: clinical severity score.

TSAb: thyroid stimulating autoantibodies.

TBII: thyroid binding inhibitory immunoglobulins.

## References

[1] Marcocci C., Bartalena L., Bogazzi F., Panicucci M., Pinchera A. (1989). Studies on the occurrence of ophthalmopathy in Graves' disease. *Acta Endocrinologica*.

[2] Bartley G. B., Gorman C. A. (1995). Diagnostic criteria for Graves' ophthalmopathy. *The American Journal of Ophthalmology*.

[3] Mourits M. P., Koornneef L., Wiersinga W. M., Prummel M. F., Berghout A., van der Gaag R. (1989). Clinical criteria for the assessment of disease activity in Graves' ophthalmology: a novel approach. *The British Journal of Ophthalmology*.

[4] Werner S. C. (1977). Modification of the classification of the eye changes of Graves' disease. *The American Journal of Ophthalmology*.

[5] Wiersinga W. M. (2012). Quality of life in Graves' ophthalmopathy. *Best Practice & Research: Clinical Endocrinology & Metabolism*.

[6] Ponto K. A., Hommel G., Pitz S., Elflein H., Pfeiffer N., Kahaly G. J. (2011). Quality of life in a German graves orbitopathy population. *The American Journal of Ophthalmology*.

[7] Bartalena L., Pinchera A., Marcocci C. (2000). Management of Graves' ophthalmopathy: reality and perspectives. *Endocrine Reviews*.

[8] Goh S. Y., Ho S. C., Seah L. L., Fong K. S., Khoo D. H. C. (2004). Thyroid autoantibody profiles in ophthalmic dominant and thyroid dominant Grave's disease differ and suggest ophthalmopathy is a multiantigenic disease. *Clinical Endocrinology*.

[9] Li Y., Kim J., Diana T., Klasen R., Olivo P. D., Kahaly G. J. (2013). A novel bioassay for anti-thyrotrophin receptor autoantibodies detects both thyroid-blocking and stimulating activity. *Clinical and Experimental Immunology*.

[10] Kamijo K., Murayama H., Uzu T., Togashi K., Olivo P. D., Kahaly G. J. (2011). Similar clinical performance of a novel chimeric thyroid-stimulating hormone receptor bioassay and an automated thyroid-stimulating hormone receptor binding assay in Graves' disease. *Thyroid*.

[11] Leschik J. J., Diana T., Olivo P. D. (2013). Analytical performance and clinical utility of a bioassay for thyroid-stimulating immunoglobulins. *The American Journal of Clinical Pathology*.

[12] Kahaly G. J., Shimony O., Gellman Y. N. (2011). Regulatory T-cells in Graves' orbitopathy: baseline findings and immunomodulation by anti-T lymphocyte globulin. *The Journal of Clinical Endocrinology and Metabolism*.

[13] Eckstein A. K., Finkenrath A., Heiligenhaus A. (2004). Dry eye syndrome in thyroid-associated ophthalmopathy: Lacrimal expression of TSH receptor suggests involvement of TSHR-specific autoantibodies. *Acta Ophthalmologica Scandinavica*.

[14] Eckstein A. K., Plicht M., Lax H. (2006). Thyrotropin receptor autoantibodies are independent risk factors for graves' ophthalmopathy and help to predict severity and outcome of the disease. *The Journal of Clinical Endocrinology and Metabolism*.

[15] Jang S. Y., Shin D. Y., Lee E. J., Choi Y. J., Lee S. Y., Yoon J. S. (2013). Correlation between TSH receptor antibody assays and clinical manifestations of Graves' orbitopathy. *Yonsei Medical Journal*.

[16] Gerding M. N., van der Meer J. W. C., Broenink M., Bakker O., Wiersinga W. M., Prummel M. F. (2000). Association of thyrotrophin receptor antibodies with the clinical features of Graves' ophthalmopathy. *Clinical Endocrinology*.

[17] Khoo D. H. C., Ho S. C., Seah L. L. (1999). The combination of absent thyroid peroxidase antibodies and high thyroid-stimulating immunoglobulin levels in Graves' disease identifies a group at markedly increased risk of ophthalmopathy. *Thyroid*.

[18] Subekti I., Boedisantoso A., Moeloek N. D., Waspadji S., Mansyur M. (2012). Association of TSH receptor antibody, thyroid stimulating antibody, and thyroid blocking antibody with clinical activity score and degree of severity of Graves ophthalmopathy. *Acta Medica Indonesiana*.

[19] Dragan L. R., Seiff S. R., Lee D. C. (2006). Longitudinal correlation of thyroid-stimulating immunoglobulin with clinical activity of disease in thyroid-associated orbitopathy. *Ophthalmic Plastic and Reconstructive Surgery*.

[20] Lytton S. D., Li Y., Olivo P. D., Kohn L. D., Kahaly G. J. (2010). Novel chimeric thyroid-stimulating hormone-receptor bioassay for thyroid-stimulating immunoglobulin. *Clinical & Experimental Immunology*.

[21] Bahn R. S. (2010). Graves' ophthalmopathy. *The New England Journal of Medicine*.

[22] Many M.-C., Costagliola S., Detrait M., Denef J.-F., Vassart G., Ludgate M. (1999). Development of an animal model of autoimmune thyroid eye disease. *Journal of Immunology*.

[23] Noh J. Y., Hamada N., Inoue Y., Abe Y., Ito K., Ito K. (2000). Thyroid-stimulating antibody is related to graves' ophthalmopathy, but thyrotropin-binding inhibitor immunoglobulin is related to hyperthyroidism in patients with Graves' disease. *Thyroid*.

[24] Bartalena L., Baldeschi L., Dickinson A. J. (2008). Consensus statement of the European group on Graves' orbitopathy (EUGOGO) on management of Graves' orbitopathy. *Thyroid*.

